# Early-onset sepsis risk calculator: a review of its effectiveness and comparative study with our evidence-based local guidelines

**DOI:** 10.1186/s13052-021-01028-1

**Published:** 2021-03-25

**Authors:** Gianluigi Laccetta, Massimiliano Ciantelli, Cristina Tuoni, Emilio Sigali, Mario Miccoli, Armando Cuttano

**Affiliations:** 1 Division of Neonatology and Neonatal Intensive Care Unit, Department of Maternal and Child Health, Santa Chiara Hospital, University of Pisa, Pisa, Italy; 2 Centro di Formazione e Simulazione Neonatale “NINA”, Azienda Ospedaliero-Universitaria Pisana, Pisa, Italy; 3 Department of Clinical and Experimental Medicine, Faculty of Medicine, University of Pisa, Pisa, Italy

**Keywords:** Early-onset sepsis, Early-onset sepsis risk calculator, Antibiotics, C-reactive protein, Procalcitonin

## Abstract

**Background:**

According to most early-onset sepsis (EOS) management guidelines, approximately 10% of the total neonatal population are exposed to antibiotics in the first postnatal days with subsequent increase of neonatal and pediatric comorbidities. A review of literature demonstrates the effectiveness of EOS calculator in reducing antibiotic overtreatment and NICU admission among neonates ≥34 weeks’ gestational age (GA); however, some missed cases of culture-positive EOS have also been described.

**Methods:**

Single-center retrospective study from 1st January 2018 to 31st December 2018 conducted in the Division of Neonatology at Santa Chiara Hospital (Pisa, Italy). Neonates ≥34 weeks’ GA with birth weight ≤ 1500 g, 34–36 weeks’ GA neonates with suspected intraamniotic infection and neonates ≥34 weeks’ GA with three clinical signs of EOS or two signs and one risk factor for EOS receive empirical antibiotics. Neonates ≥34 weeks’ GA with risk factors for EOS or with one clinical indicator of EOS undergo serial measurements of C-reactive protein and procalcitonin in the first 48–72 h of life; they receive empirical antibiotics in case of abnormalities at blood exams with one or more clinical signs of EOS. Two hundred sixty-five patients at risk for EOS met inclusion criteria; they were divided into 3 study groups: 34–36 weeks’ GA newborns (*n* = 95, group A), ≥ 37 weeks’ GA newborns (*n* = 170, group B), and ≥ 34 weeks’ GA newborns (*n* = 265, group A + B). For each group, we compared the number of patients for which antibiotics would have been needed, based on EOS calculator, and the number of the same patients we treated with antibiotics during the study period. Comparisons between the groups were performed using McNemar’s test and statistical significance was set at *p* < 0.05; post-hoc power analysis was carried out to evaluate the sample sizes.

**Results:**

32/265 (12.1%) neonates ≥34 weeks’ GA received antibiotics within the first 12 h of life. According to EOS calculator 55/265 (20.7%) patients would have received antibiotics with EOS incidence 2/1000 live births (*p* < 0.0001).

**Conclusion:**

Our evidence-based protocol entails a further decrease of antibiotic overtreatment compared to EOS calculator. No negative consequences for patients were observed.

## Background

In most high-income countries, the incidence of culture-confirmed early-onset sepsis (EOS) has decreased to 0.4–0.8 cases per 1000 live-born term infants over the last years; the overall incidence has reached about 1–2 cases per 1000 live newborns [1, 2]. This result has been achieved through a continuous update of current evidence [3–9].

As the incidence of EOS has decreased over the last two decades, clinicians raised concerns about antibiotic exposure among uninfected newborns: according to Group B Streptococcus (GBS) EOS prevention guidelines, approximately 10% of the total neonatal population are exposed to antibiotics in the first postnatal days, and almost 100% of the extremely preterm population are exposed to ampicillin and an aminoglycoside [10]. Early antibiotic exposure is associated with the emergence of antibiotic-resistant pathogenic microorganisms and with the decrease of intestinal microbial diversity, which can cause very difficult to treat infections [10]. Antibiotics administration in the neonatal period has also been linked with late onset sepsis, necrotizing enterocolitis, increased mortality and long term health outcomes such as childhood asthma, obesity, inflammatory bowel disease, celiac disease and type 1 diabetes [10]. Furthermore, administration of antibiotics to neonates often results in admission to intensive care unit, decreased breastfeeding, invasive procedures and increased hospital costs [11].

For all these reasons, it is important to avoid unnecessary antibiotics administration to patients during the early post-natal period [11]. However, the clinical diagnosis of sepsis is challenging for neonatologists because many signs of sepsis are nonspecific and are observed with other non-infectious conditions [7]. On the other side, low-level bacteremia (4 colony-forming units/mL or less), inadequate blood specimens (less than 1 mL) or maternal antibiotic treatment before or during delivery may result in negative blood cultures [1, 7]. It has been estimated that the incidence of culture-negative EOS is 6 to 16 times higher than that of culture-confirmed EOS [1]. Total white blood cell (WBC) count with its subcomponents and platelet count have also shown a poor predictive accuracy, and the specificity and selectivity of genetic biomarkers are yet to be fully evaluated [7, 12]. Protein biomarkers demonstrate high specificity and sensitivity and include C-reactive protein (CRP) and Procalcitonin (PCT), which are the most commonly used protein biomarkers for the diagnosis of sepsis and monitoring of antibiotic therapy [12–15]. Both CRP and PCT have a physiologic increase over the first 24–48 h of life; baseline concentrations of both markers are mainly affected by birth weight and gestational age (GA) [16]. On these basis, different attempts have been done to establish the appropriate cut-off values of both PCT and CRP [17–19]. Umbilical blood PCT and CRP have also been tested for EOS diagnosis; cut-off values were different among studies (0.5–2 ng/ml for PCT and 1–10 mg/l for CRP) [20].

After June 2005, several studies have assessed the safety of monitoring neonates at risk for EOS with serial physical examinations: this approach resulted in less laboratory exams and antibiotics exposure without missing any case of EOS [21–23].

In December 2012 the Kaiser Permanente EOS calculator has been developed with the purpose of avoiding antibiotic overtreatment [24]. The EOS calculator is based on a multivariate predictive risk model which allows clinicians to estimate a newborn’s individual risk for EOS given objective maternal risk factors and the infant’s clinical presentation [24]. This model permits to overcome some disadvantages of the CDC algorithm, such as the dichotomization of the continuous variables and the inclusion of maternal chorioamnionitis (CAM) as an impactful risk factor for starting antibiotic therapy [24]. A vast majority of studies about the EOS calculator demonstrates its efficacy in reducing antibiotic overtreatment, laboratory testing, painful procedures and NICU admission with increased opportunities for mother-child bonding and breastfeeding (Table 1) [11, 25–50].

**Table 1:** Summary of main articles about EOS calculator included for review

Reference	Patient population	Results
Escobar et al., 2014 [25]	≥ 34 weeks’ GA	According to 2010 CDC guidelines, 11% of infants were treated with empirical antibiotics, although only 0.04% had blood culture-confirmed sepsis. Using a risk stratification scheme based on maternal and neonatal data, 4% of infants would have been treated with empirical antibiotics
Shakib et al., 2015 [26]	≥ 34 weeks’ GA well-appearing infants exposed to maternal CAM	Reduction of patients with testing/initial antibiotics by at least 80% if using the EOS calculator compared with 2010 CDC guidelines
Kuzniewicz et al., 2017 [27]	≥ 35 weeks’ GA	Reduction of blood culture use from 14.5% (2010 CDC guidelines) to 4.9% (EOS calculator). Reduction of empiric antibiotic administration in the first 24 h from 5.0% (2010 CDC guidelines) to 2.6% (EOS calculator) with subsequent decrease of antibiotic days per 100 births from 16.0 to 8.5 days
Money et al., 2017 [28]	≥ 37 weeks’ GA well-appearing infants exposed to maternal CAM	Reduction of empiric antibiotic treatment from 99.7% (2010 CDC guidelines) to 2.5% (EOS calculator). One patient with culture-positive EOS would not have received antibiotics based on the EOS calculator
Warren et al., 2017 [29]	≥ 34 weeks’ GA infants who received antibiotics at birth for suspected EOS	Reduction of empiric antibiotic treatment from 93% (2010 CDC guidelines) to 23% (EOS calculator). Both 2010 CDC guidelines and the EOS calculator recommended treatment for 7 patients with culture-negative EOS
Beavers et al., 2018 [30]	≥ 34 weeks’ GA exposed to maternal CAM	NICU admissions rates decreased from 91 to 37%, the number of blood cultures decreased from 92 to 50% and antibiotic administration rates decreased from 94 to 37% when 2010 CDC guidelines were replaced with EOS calculator recommendations
Carola et al., 2018 [31]	≥ 35 weeks’ GA infants exposed to maternal CAM	Only 0.43% of neonates born to mothers with CAM had culture-proven EOS. Empiric antibiotics would have been recommended in 23.5% of the patients according to EOS calculator (76.5% reduction in empirical antibiotic administration compared with 2010 CDC guidelines). Blood culture only was recommended for 8.9% of the neonates; treatment with antibiotics would have been recommended for 3 of the 5 neonates with positive blood culture. All 5 neonates with positive blood cultures had abnormal CBC and CRP values at 6–12 h
Dhudasia et al., 2018 [32]	≥ 36 weeks’ GA	Reduction in antibiotics administration from 6.3 to 3.7% when current CDC guidelines were compared to EOS calculator. There was also a reduction in use of laboratory tests for suspected EOS from 26.9 to 4.9%
Gievers et al., 2018 [33]	≥ 35 weeks’ GA infants exposed to maternal CAM	Compared to the 2010 CDC guidelines, EOS calculator yields a reduction of antibiotic exposure from 95 to 9%, laboratory evaluation from 96 to 22% and NICU observation from 73 to 10%
Klingaman et al., 2018 [34]	≥ 35 weeks’ GA	Compared to the 2010 CDC guidelines, EOS calculator yields a reduction in CBCs by 88%, blood cultures by 94%, and antibiotic administration by 78%
Strunk et al., 2018 [35]	≥ 35 weeks’ GA infants requiring evaluation and/or treatment for suspected EOS	Reduction of patients admitted to NICU from 24.2 to 21.2%, decrease of blood culture sampling from 15.2 to 11.1% and reduction of empiric antibiotic administration from 12.0 to 7.6% when using EOS calculator and not local guidelines based on AAP recommendations
Akangire et al., 2019 [36]	≥ 34 weeks’ GA	Compared to current CDC/AAP guidelines, the EOS calculator-based approach yields a reduction of empiric antibiotic administration from 11.0 to 5.0% and blood culture use from 14.8 to 7.6%
Arora et al., 2019 [37]	≥ 34 weeks’ GA infants admitted to NICU	Significant reduction in the rate of both antibiotic prescriptions (70.3% vs. 49.6%) and sepsis evaluations (90.9% vs. 68.8%) after implementation of the EOS calculator. 92% overlap in blood culture recommendations and 95% overlap between antibiotic recommendations when current CDC guidelines were compared to EOS calculator
Benaim et al., 2019 [11]	≥ 34 weeks’ GA	Over the period of study, antibiotic administration decreased by 38.0% with updated local EOS guidelines. Reduction of antibiotic administration would have been 31.0% (for an EOS incidence of 0.6/1000) and 1.0% (for an EOS incidence of 2/1000) with the EOS calculator
Bridges et al., 2019 [38]	≥ 37 weeks’ GA infants exposed to maternal CAM	Compared with 2010 CDC guidelines, 93.0% of patients were not admitted to the NICU and only 11.0% required laboratory evaluation; rates of exclusive breastfeeding increased from less than 10.0% to greater than 50.0% after implementation of the EOS calculator. The length of the NICU stay decreased from an average of 138 to 12 days with no negative consequences
Eason et al., 2019 [39]	≥ 37 weeks’ GA infants with risk factors for EOS or suspected EOS	The percentage of infants screened with a suspected infection receiving 5 days of antibiotics reduced from 31.0% with NICE guidelines to 5.0% with EOS calculator. Clinically well infants with risk factors alone receiving 36 h of antibiotics, reduced from 63.0% with NICE guidelines to 3.0% with EOS calculator
Fowler et al., 2019 [40]	≥ 34 weeks’ GA	6 patients with culture-positive EOS were identified in the study period and recommendations from the calculator were in alignment with current CDC/AAP guidelines
Goel et al., 2019 [41]	≥ 34 weeks’ GA	16% of infants were started on antibiotics as per NICE recommendations compared with 4.3% with EOS calculator. There were seven positive blood cultures (three infants were recommended antibiotics by both, three were not identified in the asymptomatic stage by either; one was a contaminant)
Gong et al., 2019 [42]	≥ 34 weeks’ GA infants exposed to maternal intrapartum fever	Compared to the CDC/AAP guidelines, the EOS calculator-based approach yields a net monetary benefit (3998 $ per infant), largely by preventing unnecessary antibiotic treatment (67.4% decrease in antibiotic use in the calculator arm)
Hershkovich-Shporen et al., 2019 [43]	≥ 35 weeks’ GA newborns with the following inclusion criteria: treated with antibiotic, born to mothers with risk factors for EOS, born to mothers with clinical CAM or that received IAP	15.0% of the patients received antibiotic treatment according to 2010 CDC recommendations; 8.0% of the patients would have received antibiotic treatment according to EOS calculator. Only 2/89 (2.25%) newborns treated for maternal clinical CAM according to 2010 CDC guidelines, had proven EOS. Three of the mothers whose newborn developed EOS, had no risk factors so there was no need for the EOS calculator
Joshi et al., 2019 [44]	≥ 34 weeks’ GA well-appearing newborns exposed to maternal CAM	Compared to the CDC/AAP guidelines, the usage of the EOS calculator yields a reduction of empirical antibiotics administration from 100% of patients to 8.9%
Leonardi et al., 2019 [45]	≥ 35 weeks’ GA newborns exposed to maternal CAM and/or intrapartum fever	228/312 (73.1%) infants did not require admission to the NICU based on their risk assessment using the EOS calculator; according to local guidelines, all infants would have been admitted to the NICU for evaluation and treatment of presumed sepsis, regardless of clinical appearance. Breastfeeding rates at discharge were 89.0% for infants remaining with their mothers in the newborn nursery, and 37.0% for infants admitted to the NICU
Stipelman et al., 2019 [46]	≥ 34 weeks’ GA infants exposed to maternal CAM	Reduction in antibiotics administration from 7.0% (according to CDC/AAP guidelines) to 1.0% after implementation of the EOS calculator. 2 missed cases of culture-positive EOS with EOS calculator
Benincasa et al., 2020 [47]	≥ 34 weeks’ GA neonates who received EOS antibiotics according to the hospital’s current practice	219/384 (57.0%) patients received antibiotics by EOS calculator and 64/384 (16.7%) by evaluation of clinical signs. All patients with positive blood culture were detected by both EOS calculator and clinical signs surveillance. Estimated costs were US$ 415.576 for EOS calculator and US$ 314.353 for evaluation of clinical signs
Morris et al., 2020 [48]	≥ 34 weeks’ GA infants with EOS confirmed on blood or cerebrospinal fluid culture	Within 4 h of birth, antibiotics were recommended for 39/70 (55.7%) infants with NICE guidelines, compared with 27/70 (38.6%) with the EOS calculator. The 12 infants advised early treatment only by NICE guidelines remained well, only one showing mild symptoms after 4 h. Another 4 babies received antibiotics by 4 h outside NICE and EOS calculator guidance. The remaining 27 infants (38.6%) received antibiotics when symptomatic after 4 h. Only one infant who was unwell from birth, died. Both NICE guidelines and EOS calculator were poor in identifying EOS within 4 h; NICE guidelines were superior to the EOS calculator in identifying asymptomatic cases
Perez et al., 2020 [49]	≥ 35 weeks’ GA	Compared to the current AAP guidelines, the usage of the EOS calculator yields 54.0% reduction in the number of infants undergoing sepsis workup evaluations and 51.0% decrease in the number of infants receiving antibiotics
van der Weijden et al., 2020 [50]	≥ 34 weeks’ GA neonates at risk for EOS	Dutch guidelines recommended antibiotic treatment for 363/890 (40.8%) neonates versus 101/890 (11.3%) with EOS calculator (*p* < 0.01). Antibiotic treatment was recommended by both methods for 90/890 (10.1%) neonates, including 2 patients with positive blood culture

*CAM* Chorioamnionitis, *CBC* Cell blood count, *CRP* C-reactive protein, *EOS* Early-onset sepsis, *GA* Gestational age, *IAP* Intrapartum antibiotic prophylaxis

The objective of our study was to compare the administration of antibiotics based on our local EOS guidelines derived from current evidence with the calculator’s recommendations in neonates born at ≥34 weeks’ GA.

## Methods

This was a single-center retrospective study from 1st January 2018 to 31st December 2018 conducted in the Division of Neonatology at Santa Chiara Hospital (Pisa, Italy). The parents of all subjects signed a written consent form and the study was approved by the ethics committee of the Meyer Children’s Hospital of Florence. Based on our local guidelines, neonates born at ≥34 weeks’ GA are divided into three categories (high, medium and low EOS risk) as shown in Table 2, and managed as shown in Table 3.

**Table 2:** EOS risk categories for neonates born at ≥34 weeks’ GA

EOS risk categories	Included patients
High-risk patients	≥ 34 weeks’ GA neonates with birth weight ≤ 1500 g
	34–36 weeks’ GA neonates with suspected intraamniotic infection
	≥ 34 weeks’ GA neonates with three clinical signs of EOS
	≥ 34 weeks’ GA neonates with two clinical signs and one risk factor for EOS
Medium-risk patients	34 weeks’ GA neonates without suspicion of intraamniotic infection
	≥ 35 weeks’ GA neonates from mothers with previous infant affected by invasive GBS disease and inadequate IAP^a^
	≥ 35 weeks’ GA neonates from mothers with GBS bacteriuria during any trimester of the current pregnancy and inadequate IAP (not if a cesarean delivery is performed before onset of labor on a woman with intact amniotic membranes)^a^
	≥ 35 weeks’ GA neonates from mothers with positive GBS vaginal-rectal screening culture within 5 weeks before delivery and inadequate IAP (not if a cesarean delivery is performed before onset of labor on a woman with intact amniotic membranes)^a^
	35–36 weeks’ GA neonates with unknown GBS maternal status at the onset of labor and inadequate IAP (not if a cesarean delivery is performed before onset of labor on a woman with intact amniotic membranes)^a^
	≥ 35 weeks’ GA neonates from mothers with amniotic membrane rupture ≥18 h and inadequate IAP^a^
	35–36 weeks’ GA neonates from mothers with intrapartum temperature ≥ 38.0 °C
	≥ 37 weeks’ GA neonates with suspected intraamniotic infection and inadequate IAP^a^
	≥ 37 weeks’ GA neonates with maternal intrapartum temperature ≥ 38.0 °C and inadequate IAP^a^
	≥ 34 weeks’ GA neonates with one or two clinical indicators of EOS
Low-risk patients	Well-appearing neonates ≥34 weeks’ GA with no risk factors for EOS

^a^Screening for vaginal-rectal GBS colonization and use of IAP are based on CDC 2010 guidelines [5]; however, at our institution, intrapartum intravenous ampicillin or cefazolin (1 g every 8 h until delivery) is also administered in case of amniotic membrane rupture ≥18 h and negative vaginal-rectal screening culture. IAP is considered adequate when intravenous penicillin, ampicillin or cefazolin is administered ≥4 h before delivery, in accordance with CDC 2010 guidelines [5]

*EOS* Early-onset sepsis, *GA* Gestational age, *GBS* Group B Streptococcus, *IAP* Intrapartum antibiotic prophylaxis

**Table 3:** Management of newborns ≥34 weeks’ GA according to our local guidelines

EOS risk categories	Management
High-risk patients	Full diagnostic evaluation^ab^ and empirical antibiotics^c^ pending the results of the evaluation
	CRP and PCT at 24 ± 4 h of life^b^
	CRP and PCT at 48 ± 4 h of life^b^
	CRP and PCT at 72 ± 4 h of life^b^
Medium-risk patients	Limited diagnostic evaluation^db^
	CRP and PCT at 24 ± 4 h of life^b^
	CRP and PCT at 48 ± 4 h of life^b^
	CRP and PCT at 72 ± 4 h of life^eb^
	Further exams (CBC, blood culture)^b^ and empirical antibiotics^c^ in presence of one clinical indicator of EOS and one of the following conditions: 1) Abnormal cord blood PCT^b^; 2) Abnormal neonatal PCT before 28 h of life^b^; 3) Abnormal neonatal CRP and PCT after 28 h of life^b^
Low-risk patients	Routinely observation for ≥48 h before discharge

**Fig. 1:**
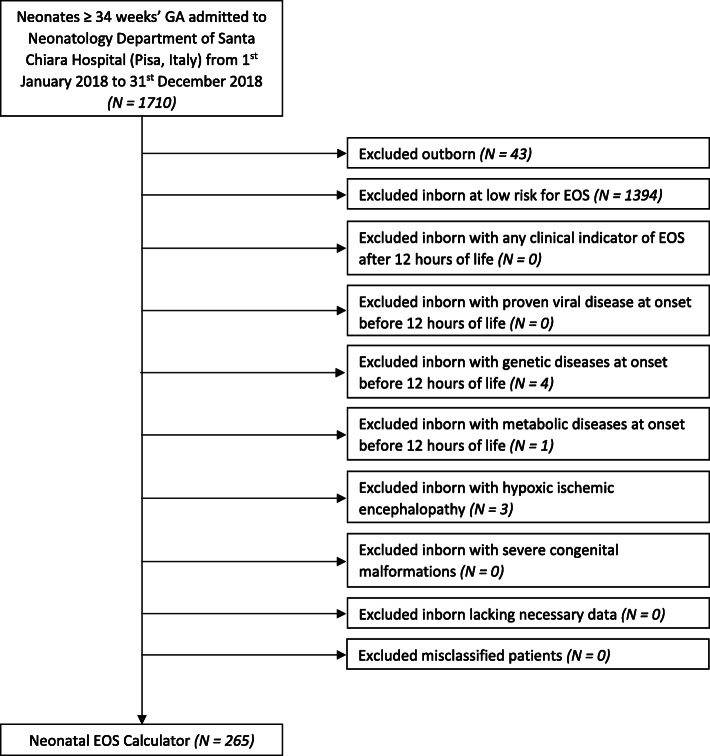
Selection process of the study population. Legends: EOS, early-onset sepsis; GA, gestational age

The whole study population included all newborns ≥34 weeks’ GA consecutively admitted to the Neonatology Department of Santa Chiara Hospital (Pisa, Italy) during the study period. The selection process of the study participants is represented in Fig. 1; the study groups included all newborns ≥34 weeks’ GA, newborns 34–36 weeks’ GA, and newborns ≥37 weeks’ GA. All included patients have been managed in accordance with our local guidelines

*CBC* Cell blood count, *CRP* C-reactive protein, *EOS* Early-onset sepsis, *GA* Gestational age, *PCT* Procalcitonin

^a^Cord blood CRP and PCT or measurement of both markers within the first hour of life or at the onset of symptoms, blood culture and complete blood count (CBC) before receiving empirical antibiotics

^b^The quantities of blood used for laboratory analyses are the following ones: 200 μL for CRP or PCT, 300 μL for both CRP and PCT, 400 μL for CBC and 1 mL for blood culture. Measurement of CRP and/or PCT is also possible on capillary blood samples. Cord blood CRP ≥ 10 mg/L and cord blood PCT ≥ 0.6 ng/mL are considered pathological; CRP ≥ 10 mg/L is considered abnormal even when performed on neonatal blood samples. PCT requires adjustment of the cut-off point with time according to the age-specific 95% reference intervals by Chiesa et al., when performed on neonatal blood samples [17]

^c^Intravenous ampicillin-sulbactam and gentamicin. Prophylaxis with empirical antibiotics is interrupted at 72 ± 4 h of life in asymptomatic patients with negative blood culture and normal neonatal CRP and PCT. Antibiotic treatment is continued for another 4–11 days, for a total of 7–14 days, in the following cases: 1) Patients with clinical indicators of EOS at 72 ± 4 h of life; 2) Abnormal CRP and/or PCT at 72 ± 4 h of life; 3) Positive blood culture

^d^Cord blood CRP and PCT or measurement of both markers within the first hour of life or at the onset of symptoms

^e^Only symptomatic patients and preterm newborns

Neonates born at ≥34 weeks’ GA during the study period at our institution were identified using admission logs. Thus, we retrospectively reviewed maternal and neonatal charts and collected data for input into the EOS calculator. We also collected data about other risk factors for EOS, mode of delivery, duration of labor, presence and type of clinical indicators of EOS with the time in which they appeared, relevant laboratory results, type and duration of antibiotic therapy. These data were obtained in order to verify physicians’ compliance with our local guidelines and the correct classification of medium- and high-risk patients into non-septic patients, patients with culture-positive EOS and patients with culture-negative EOS. Thus, we calculated both culture-positive EOS and culture-negative EOS plus culture-positive EOS incidence rates at our institution during the study period. No cases of culture-positive EOS were observed among the study population; 4 cases of culture-negative EOS were reported among inborn infants ≥34 weeks’ GA. All 4 patients with culture-negative EOS had no risk factors for EOS and were medium-risk patients ≥37 weeks’ GA with one or two clinical signs of EOS within the first 12 h of life. They all presented simultaneous increase of both CRP and PCT at the onset of symptoms or increase of PCT at the onset of symptoms followed by an increase of CRP. Thus, the incidence of culture-negative EOS plus culture-positive EOS among inborn infants ≥34 weeks’ GA during the study period was 2.4/1000 live births. Thereafter, misclassified patients have been excluded from the study (Fig. 1). We then classified each patient as well appearing, equivocal, or with clinical illness as specified on the Kaiser Permanente website (https://neonatalsepsiscalculator.kaiserpermanente.org). Each patient’s EOS risk and subsequent management recommendation were determined using the EOS calculator with culture-positive EOS incidence rate (approximated at 0.1/1000 live births); we also re-calculated EOS risk and management recommendation for each patient based on culture-negative EOS plus culture-positive EOS incidence rate (approximated at 2/1000 live births). Possible management recommendations were as follows: 1) No culture, no antibiotics, routine vitals; 2) No culture, no antibiotics, vitals every 4 h for 24 h; 3) Blood culture, vitals every 4 h for 24 h; 4) Strongly consider starting empiric antibiotics, vitals per NICU; 5) Empiric antibiotics, vitals per NICU. We recorded all management recommendations and classified them into 2 categories, as shown in Table 4.

**Table 4:** Classification of EOS calculator’s management recommendations according to our study protocol

EOS calculator’s management recommendations		Our study protocol
No. 1	No culture, no antibiotics, routine vitals	No antibiotics needed (A)
No. 2	No culture, no antibiotics, vitals every 4 h for 24 h	
No. 3	Blood culture, vitals every 4 h for 24 h	
No. 4	Strongly consider starting empiric antibiotics, vitals per NICU	Antibiotics needed (B)
No. 5	Empiric antibiotics, vitals per NICU	

*EOS* Early-onset sepsis, *NICU* Neonatal intensive care unit

For each study group, we compared the number of patients for which antibiotics would have been needed, based on EOS calculator, and the number of the same patients we treated with antibiotics during the study period. Data were collected into a designated database. We therefore used R Software version 3.6.2 for statistical evaluations; comparisons between the groups were performed using McNemar’s test for paired nominal data and statistical significance was set at *p* < 0.05. Post-hoc power analysis was carried out to evaluate the sample sizes.

## Results

A total of 1667 neonates born at ≥34 weeks’ GA during the study period at our institution were identified using admission logs. Patients at low risk for EOS (1394/1667, 83.6%) and those who met exclusion criteria (8/1667, 0.5%) were excluded from the study. Thus, a total of 265 (15.9%) patients fulfilled inclusion criteria and were enrolled in the study. Demographic characteristics and risk factors for EOS of the study subjects are shown in Table 5.

**Table 5:** Demographic characteristics and risk factors for EOS of the study subjects (*n* = 265)

Characteristics	34 weeks’ GA (*n* = 27)	35–36 weeks’ GA (*n* = 68)	≥ 37 weeks’ GA (*n* = 170)
Birth weight (range in kg)	1.56–2.86	1.39–3.82	1.51–4.49
Birth weight ≤ 1500 g, n (%)	0 (0.0)	2 (2.9)	0 (0.0)
SGA, n (%)	0 (0.0)	6 (8.8)	7 (4.1)
Gender, n (%)			
Male	19 (70.4)	36 (52.9)	100 (58.8)
Female	8 (29.6)	32 (47.1)	70 (41.2)
Mode of delivery, n (%)			
Vaginal	6 (22.2)	12 (17.6)	132 (77.6)
Cesarean section	21 (77.8)	56 (82.4)	38 (22.4)
Multiple births/Total births, (%)	8/19 (42.1)	11/56 (19.6)	1/169 (0.6)
APGAR ≥7 at 5 min, n (%)	27 (100.0)	66 (97.1)	170 (100.0)
GBS status at birth, n (%)			
Negative	15 (55.6)	23 (33.8)	59 (34.7)
Positive	2 (7.4)	12 (17.6)	92 (54.1)
Unknown	10 (37.0)	33 (48.5)	19 (11.2)
Duration of ROM (range in hours)	0–235	0–219	0–117
ROM ≥18 h, n (%)	4 (14.8)	11 (16.2)	37 (21.8)
IAP, n (%)			
Adequate	4 (14.8)	12 (17.6)	23 (13.5)
Inadequate	23 (85.2)	56 (82.4)	147 (86.5)
Previous infant affected by invasive GBS disease, n (%)	0 (0.0)	0 (0.0)	0 (0.0)
GBS bacteriuria during any trimester of the current pregnancy, n (%)	0 (0.0)	3 (4.4)	6 (3.5)
Maternal intrapartum temperature ≥ 38.0 °C, n (%)	0 (0.0)	0 (0.0)	4 (2.3)
Suspected intraamniotic infection, n (%)	0 (0.0)	1 (1.5)	3 (1.8)
GBS bacteriuria during any trimester of the current pregnancy and inadequate IAP^a^, n (%)	0 (0.0)	1 (1.5)	5 (2.9)
Positive GBS vaginal-rectal screening culture within 5 weeks before delivery and inadequate IAP^a^, n (%)	0 (0.0)	3 (4.4)	77 (45.3)
Unknown GBS maternal status at the onset of labor and inadequate IAP^a^, n (%)	4 (14.8)	8 (11.8)	17 (10.0)
ROM ≥18 h and inadequate IAP, n (%)	0 (0.0)	3 (4.4)	24 (14.1)
Suspected intraamniotic infection and inadequate IAP, n (%)	0 (0.0)	1 (1.5)	1 (0.6)
Maternal intrapartum temperature ≥ 38.0 °C and inadequate IAP, n (%)	0 (0.0)	0 (0.0)	2 (1.2)
Neonates with three clinical signs of EOS, n (%)	0 (0.0)	0 (0.0)	2 (1.2)
Neonates with two clinical signs and one risk factor for EOS, n (%)	13 (48.1)	8 (11.8)	22 (12.9)
Neonates with one or two clinical indicators of EOS, n (%)	0 (0.0)	50 (73.5)	59 (34.7)
High-risk patients, n (%)	13 (48.1)	11 (16.2)	24 (14.1)
Medium-risk patients, n (%)	14 (51.9)	57 (83.8)	146 (85.9)

*EOS* Early-onset sepsis, *GA* Gestational age, *GBS* Group B Streptococcus, *IAP* Intrapartum antibiotic prophylaxis, *ROM* Rupture of membranes, *SGA* Small for gestational age

^a^Not if a cesarean delivery is performed before onset of labor on a woman with intact amniotic membranes

According to our guidelines, 32/265 (12.1%) neonates were initiated on antibiotics in the first 12 h of life; none was initiated on antibiotics at 13–72 h of life.

**Fig. 2:**
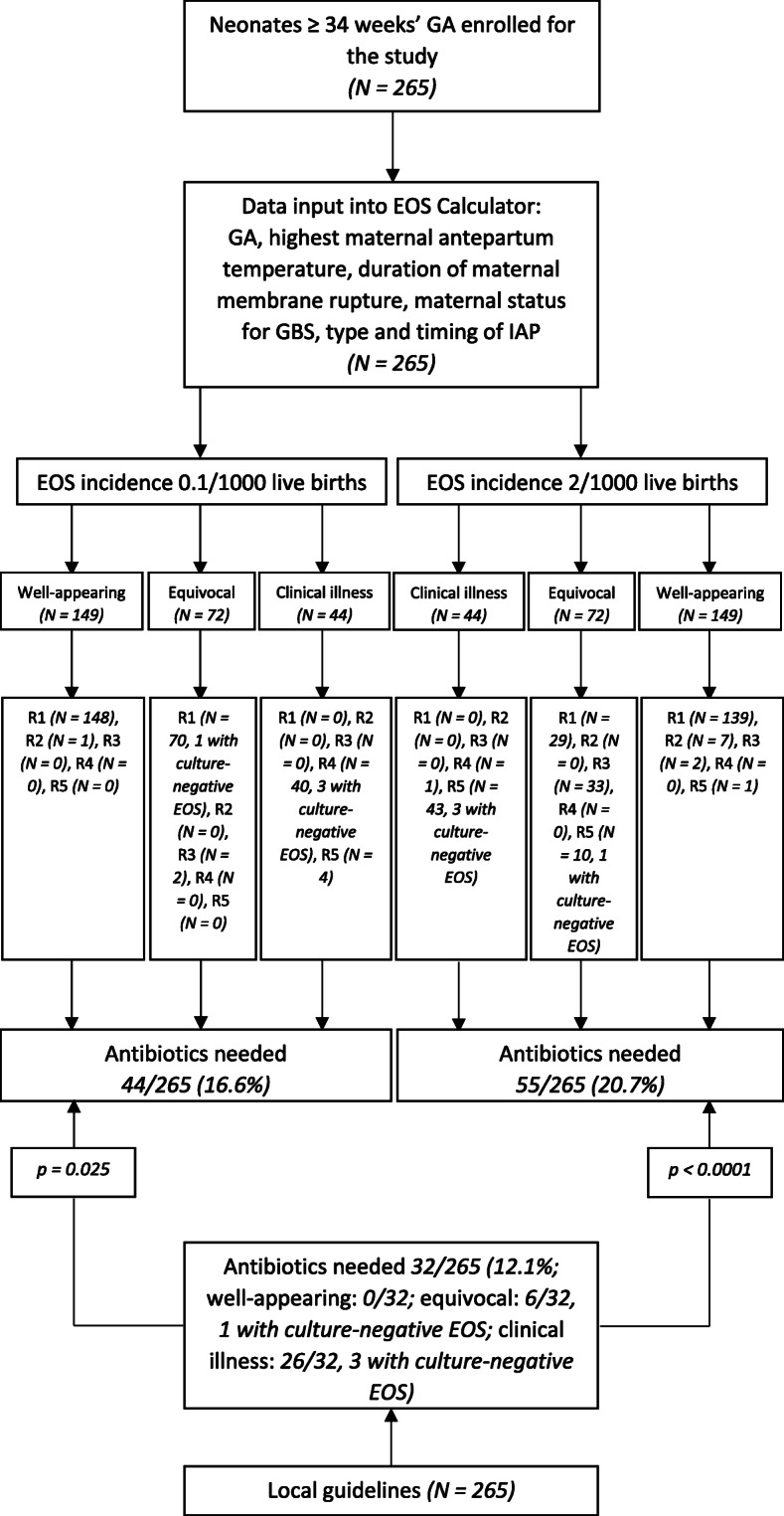
Comparison between our local guidelines and EOS calculator. Neonates ≥34 weeks’ GA. Legends: EOS, early-onset sepsis; GA, gestational age; GBS, Group B Streptococcus; IAP, intrapartum antibiotic prophylaxis; R1, recommendation No. 1 (No culture, no antibiotics, routine vitals); R2, recommendation No. 2 (No culture, no antibiotics, vitals every 4 h for 24 h); R3, recommendation No. 3 (Blood culture, vitals every 4 h for 24 h); R4, recommendation No. 4 (Strongly consider starting empiric antibiotics, vitals per NICU); R5, recommendation No. 5 (Empiric antibiotics, vitals per NICU)

After entering the data into the EOS calculator with local EOS incidence of 2/1000 live births, the recommendations were as follows: 1) No culture, no antibiotics, routine vitals (168 patients); 2) No culture, no antibiotics, vitals every 4 h for 24 h (7 patients); 3) Blood culture, vitals every 4 h for 24 h (35 patients); 4) Strongly consider starting empiric antibiotics, vitals per NICU (1 patient); 5) Empiric antibiotics, vitals per NICU (54 patients). Thus, according to EOS calculator, antibiotics were needed in 55/265 (20.7%) patients in the first 12 h of life. The difference with our local guidelines resulted statistically significant (*p* < 0.0001). Data are shown in Fig. 2.

As no cases of culture-positive EOS were observed during the study period, we also entered the same data into the EOS calculator with the lowest possible local EOS incidence (0.1/1000 live births). The recommendations were as follows: 1) No culture, no antibiotics, routine vitals (218 patients); 2) No culture, no antibiotics, vitals every 4 h for 24 h (1 patient); 3) Blood culture, vitals every 4 h for 24 h (2 patients); 4) Strongly consider starting empiric antibiotics, vitals per NICU (40 patients); 5) Empiric antibiotics, vitals per NICU (4 patients). Thus, according to EOS calculator, antibiotics were needed in 44/265 (16.6%) patients in the first 12 h of life; the difference with our local guidelines resulted statistically significant even in this case (*p* < 0.025).

A full-term newborn with culture-negative EOS starting with respiratory distress 6 h after birth received antibiotics according to our local guidelines; when using EOS calculator, this patient was classified as “equivocal” and would not have received antibiotics with EOS incidence 0.1/1000 live births.

As regards treatment, overlap between EOS calculator recommendations and our local guidelines was 88.3% (234/265 patients) when using EOS calculator with EOS incidence 2/1000 live births, and 90.9% (241/265 patients) when using EOS calculator with EOS incidence 0.1/1000 live births. Data are shown in Fig. 2.

The patients enrolled in the study were hence assessed by dividing them into 2 groups: 1) 34–36 weeks’ GA neonates; 2) ≥ 37 weeks’ GA neonates.

**Fig. 3:**
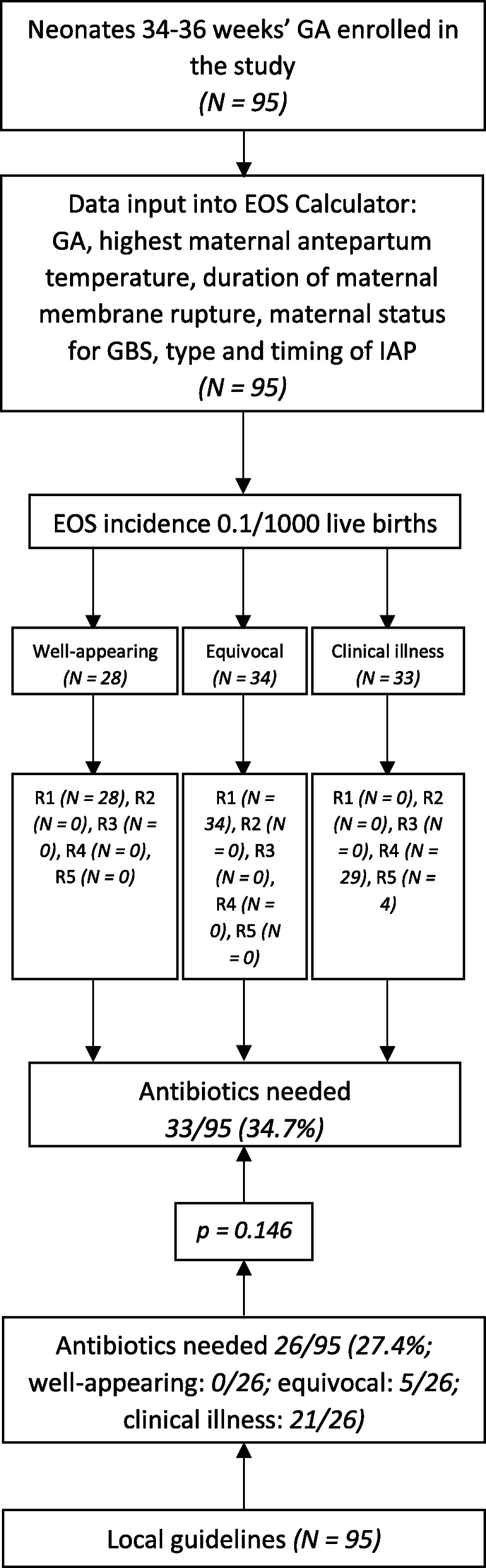
Comparison between our local guidelines and EOS calculator. Neonates 34–36 weeks’ GA. Legends: EOS, early-onset sepsis; GA, gestational age; GBS, Group B Streptococcus; IAP, intrapartum antibiotic prophylaxis; R1, recommendation No. 1 (No culture, no antibiotics, routine vitals); R2, recommendation No. 2 (No culture, no antibiotics, vitals every 4 h for 24 h); R3, recommendation No. 3 (Blood culture, vitals every 4 h for 24 h); R4, recommendation No. 4 (Strongly consider starting empiric antibiotics, vitals per NICU); R5, recommendation No. 5 (Empiric antibiotics, vitals per NICU)

Inborn infants 34–36 weeks’ GA were 95/265 (35.8%). According to our local guidelines, 26/95 (27.4%) of these neonates were initiated on antibiotics in the first 12 h of life. Neither culture-positive nor culture-negative EOS were observed among infants 34–36 weeks’ GA during the study period. After entering data into the EOS calculator with the lowest possible local EOS incidence (0.1/1000 live births), the recommendations for patients 34–36 weeks’ GA were as follows: 1) No culture, no antibiotics, routine vitals (62 patients); 2) No culture, no antibiotics, vitals every 4 h for 24 h (0 patients); 3) Blood culture, vitals every 4 h for 24 h (0 patients); 4) Strongly consider starting empiric antibiotics, vitals per NICU (29 patients); 5) Empiric antibiotics, vitals per NICU (4 patients). Thus, according to EOS calculator, antibiotics were needed in 33/95 (34.7%) patients 34–36 weeks’ GA in the first 12 h of life; the difference with our local guidelines was not statistically significant (*p* = 0.146), although 7 more patients would have been treated using EOS calculator compared to our approach. Data are shown in Fig. 3.

**Fig. 4:**
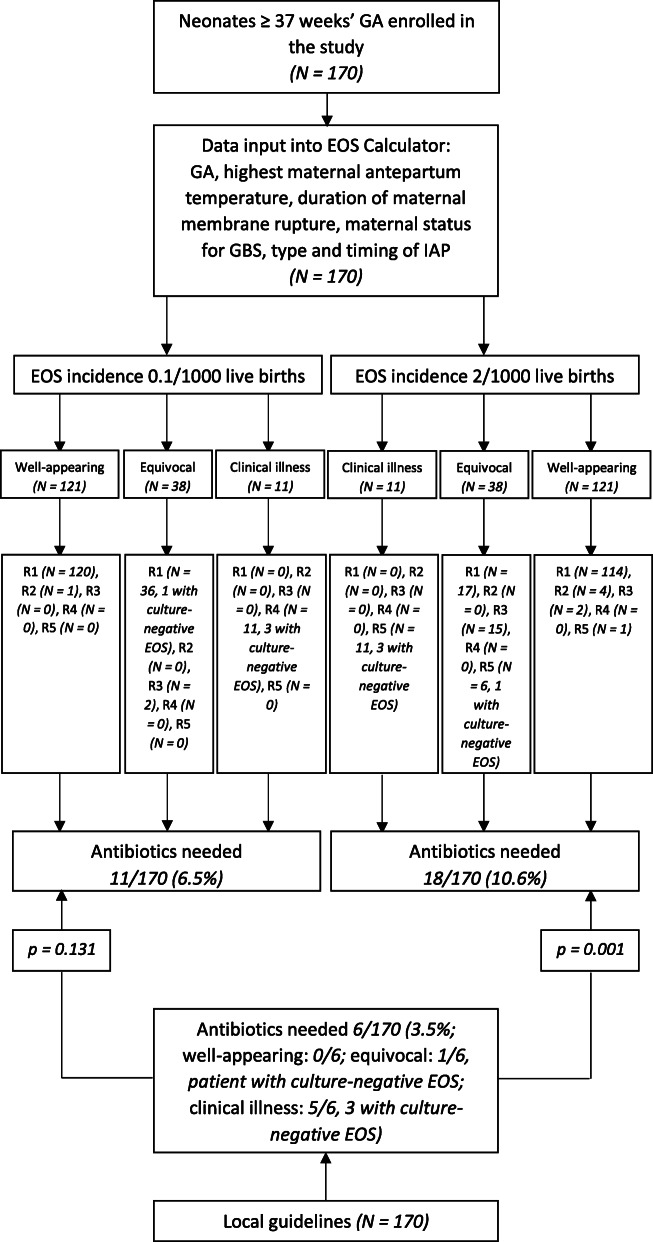
Comparison between our local guidelines and EOS calculator. Neonates ≥37 weeks’ GA. Legends: EOS, early-onset sepsis; GA, gestational age; GBS, Group B Streptococcus; IAP, intrapartum antibiotic prophylaxis; R1, recommendation No. 1 (No culture, no antibiotics, routine vitals); R2, recommendation No. 2 (No culture, no antibiotics, vitals every 4 h for 24 h); R3, recommendation No. 3 (Blood culture, vitals every 4 h for 24 h); R4, recommendation No. 4 (Strongly consider starting empiric antibiotics, vitals per NICU); R5, recommendation No. 5 (Empiric antibiotics, vitals per NICU)

Inborn infants ≥37 weeks’ GA were 170/265 (64.2%). According to our local guidelines, 6/170 (3.5%) of these neonates were initiated on antibiotics in the first 12 h of life. A retrospective analysis of blood culture, CRP and PCT results showed no cases of culture-positive EOS and 4 cases of culture-negative EOS among the 1532 inborn infants ≥37 weeks’ GA during the study period. Thus, the calculated incidence rate of EOS was 2.6/1000 live births. After entering data into the EOS calculator with local EOS incidence of 2/1000 live births, the recommendations were as follows: 1) No culture, no antibiotics, routine vitals (131 patients); 2) No culture, no antibiotics, vitals every 4 h for 24 h (4 patients); 3) Blood culture, vitals every 4 h for 24 h (17 patients); 4) Strongly consider starting empiric antibiotics, vitals per NICU (0 patients); 5) Empiric antibiotics, vitals per NICU (18 patients). Thus, according to EOS calculator, antibiotics were needed in 18/170 (10.6%) patients in the first 12 h of life; the difference with our local guidelines resulted statistically significant (*p* = 0.001). Data are shown in Fig. 4.

As no cases of culture-positive EOS were observed among inborn infants ≥37 weeks’ GA, we also entered the same data into the EOS calculator with the lowest possible local EOS incidence (0.1/1000 live births). The recommendations were as follows: 1) No culture, no antibiotics, routine vitals (156 patients); 2) No culture, no antibiotics, vitals every 4 h for 24 h (1 patient); 3) Blood culture, vitals every 4 h for 24 h (2 patients); 4) Strongly consider starting empiric antibiotics, vitals per NICU (11 patients); 5) Empiric antibiotics, vitals per NICU (0 patients). Thus, according to EOS calculator, antibiotics were needed in 11/170 (6.5%) patients in the first 12 h of life; the difference with our local guidelines was not statistically significant (*p* = 0.131), although 5 more patients would have been treated using EOS calculator compared to our approach. Data are shown in Fig. 4.

Post-hoc power analysis for statistically significant differences revealed that sample sizes were appropriate.

## Discussion

Early diagnosis and treatment decision-making of neonatal EOS are challenging for clinicians; at the same time antibiotic resistance is an increasing problem, thus antibiotic overexposure among neonates should be avoided. For this purpose, we revisited the antibiotic stewardship program at our institution and drew up a protocol for management of neonates at risk for EOS.

The neonatal EOS calculator has been introduced to support the clinician’s treatment decision-making of neonatal EOS. Most of Authors agree on the efficacy of the EOS calculator in reducing antibiotic overtreatment; however, some Authors have also reported patients with culture-positive EOS who would not have received antibiotics based on the EOS calculator. In our study, according to our local guidelines, antibiotics were needed in 32/265 enrolled patients ≥34 weeks’ GA in the first 12 h of life; based on the EOS calculator, antibiotics would have been needed in 44/265 patients when using an EOS incidence of 0.1/1000 live births, and in 55/265 patients when using an EOS incidence of 2/1000 live births. As both differences resulted statistically significant, the use of our protocol is advantageous in clinical practice.

According to our study, a missed case of culture-negative EOS was observed when using the EOS calculator with EOS incidence 0.1/1000 live births; however, this EOS incidence includes only culture-positive EOS cases and, probably, underrates the true incidence of EOS at our institution.

Furthermore, when using the EOS calculator all patients classified as “clinical illness” would have received antibiotics regardless of EOS incidence; according to our local guidelines 26/44 of these neonates received antibiotics with no negative consequences.

We think that the effectiveness of our protocol results from the inclusion of anamnestic data, clinical evaluation and laboratory exams. Anamnestic data permitted us to identify neonates with risk factors for EOS or elements which could explain clinical presentation (for example gestational diabetes, meconium aspiration or short labor in patients with respiratory distress). Thus, not all neonates classified as “clinical illness” received antibiotics according to our protocol. Clinical evaluation is very important since none of the patients with culture-negative EOS had risk factors for EOS, thus they were identified for the presence of clinical signs. Clinical evaluation is even crucial to decide whether to start antibiotic therapy because of the possibility of false-negatives with blood culture and false-positives with measurement of CRP and PCT. However, serial CRP and PCT measurements allowed us to identify patients with EOS, to control the efficacy of antibiotic therapy, and to decide when to stop antibiotic treatment. We think EOS calculator is an effective tool to reduce unnecessary antibiotics administration to neonates but it also has several limitations. First, the highest possible EOS incidence is 4/1000 live births, thus EOS calculator cannot be utilized in contexts with EOS incidence higher than 4/1000 live births. Second, its use is limited in the first 12 h of life but EOS can manifest itself between 12 and 72 h of life, although rarely, and serial measurements of CRP and PCT in the first 72 h of life allow us to identify all cases of EOS. Third, antibiotics are indicated to all neonates classified as “clinical illness” (persistent need for nCPAP/HFNC/mechanical ventilation outside of the delivery room, hemodynamic instability requiring vasoactive drugs, neonatal encephalopathy/perinatal depression, need for supplemental O_2_ ≥ 2 h to maintain oxygen saturations > 90% outside of the delivery room); we think that careful consideration of risk factors for EOS, anamnestic data and alternative diagnoses should further reduce unnecessary antibiotics administration. Fourth, equivocal patients can present with tachycardia, tachypnea, temperature instability or respiratory distress; however other clinical indicators of possible EOS (altered behaviour or responsiveness, feeding difficulties etc.) should be considered. Fifth, laboratory exams should be considered to reduce the number of patients receiving unnecessary antibiotics, above all among patients classified as “clinical illness”, and to identify patients with EOS appearing after 12 h of life. Furthermore, our protocol incorporates the new definition for CAM: this disease is now defined as intraamniotic infection or “Triple I” and requires more clinical features for diagnosis. Thus, we make difference between neonates from mothers with “Triple I” and those with isolated maternal fever: this contributes in reducing the number of neonates receiving antibiotics.

However, even our protocol has many limitations. First, the number of 34–36 weeks’ GA neonates receiving antibiotics is too high (26/95, none with EOS). Thus, we should re-evaluate clinical criteria for starting antibiotics and the optimal cut-off point for both CRP and PCT in late-preterm infants. Birth weight ≤ 1500 g should also be re-evaluated as a criteria to start antibiotics. Second, reducing antibiotics administration is money-saving. However, laboratory exams, above all PCT, are quite expensive. Third, we need serial clinical evaluations to identify neonates with clinical signs of EOS, especially those without maternal risk factors. However, even applying the EOS calculator requires serial clinical evaluations in the first 12 h of life. Fourth, serial blood samplings are needed for measurement of CRP and PCT; however, the first measurement is usually performed on cord blood, and blood sampling at 48 ± 4 h of life is the same for the newborn screening test. The remaining measurements sometimes coincide with blood samplings for gas analysis or glycemia evaluation. Fifth, our study is retrospective. Even if the course of each patient is well documented, the classification of neonates into well-appearing, equivocal or clinically ill is partly dependent on whomever is analyzing the medical records. Sixth, we should consider an earlier interruption of antibiotics at 48 h of life in well-appearing neonates with negative laboratory exams in order to reduce both antibiotics exposure and laboratory exams. Seventh, the EOS calculator has already been validated on more than 180.000 newborns, thus we should also validate our protocol on large scale to definitively prove its superiority.

## Conclusion

EOS calculator has been proven to be an effective tool for treatment decision-making of neonatal EOS, however we have shown a further decrease in antibiotics administration through a continuous evidence-based update of local guidelines. Thus, continuous review of recommendations and updated guidelines are necessary to reduce both antibiotics administration and microbial resistance, with consequent reduction of related comorbidities, and to pursue the best possible antibiotic stewardship.

## Data Availability

The datasets used and analyzed during the current study are available from the corresponding author on reasonable request.

## Abbreviations

CAM: Maternal chorioamnionitis
CBC: Complete blood count
CRP: C-reactive protein
EOS: Early-onset sepsis
GA: Gestational age
GBS: Group B Streptococcus
IAP: Intrapartum antimicrobial prophylaxis
NICU: Neonatal intensive care unit
PCT: Procalcitonin
WBC: White blood cell

## References

[1] Klingenberg C, Kornelisse RF, Buonocore G, Maier RF, Stocker M (2018). Culture-negative early-onset neonatal Sepsis – at the crossroad between efficient sepsis care and antimicrobial stewardship. Front Pediatr.

[2] Cortese F, Scicchitano P, Gesualdo M, Filaninno A, De Giorgi E, Schettini F, Laforgia N, Ciccone MM (2016). Early and late infections in newborns: where do we stand? A review. Pediatr Neonatol.

[3] Centers for Disease Control and Prevention (1996). Prevention of perinatal Group B Streptococcal disease: a public health perspective. MMWR Recomm Rep.

[4] Schrag S, Gorwitz R, Fultz-Butts K, Schuchat A (2002). Prevention of perinatal Group B Streptococcal disease. Revised guidelines from CDC. MMWR Recomm Rep.

[5] Verani JR, McGee L, Schrag SJ, Division of Bacterial Diseases, National Center for Immunization and Respiratory Diseases, Centers for Disease Control and Prevention (CDC) (2010). Prevention of perinatal Group B Streptococcal disease. Revised guidelines from CDC 2010. MMWR Recomm Rep.

[6] The American College of Obstetricians and Gynecologists (2011). Committee on Obstetric Practice. ACOG Committee Opinion No.485. Prevention of early-onset group B streptococcal disease in newborns. Obstet Gynecol.

[7] Polin RA, the Committee on fetus and newborn (2012). Management of neonates with suspected or proven early-onset bacterial sepsis. Pediatrics.

[8] National Institute for Health and Clinical Excellence. Neonatal infection (early onset): antibiotics for prevention and treatment (CG149). Clinical guideline. 2012. Available online at www.nice.org.uk/guidance/cg149.

[9] Brady MT, Polin RA (2013). Prevention and management of infants with suspected or proven neonatal sepsis. Pediatrics.

[10] Cotten MC (2016). Adverse consequences of neonatal antibiotic exposure. Curr Opin Pediatr.

[11] Benaim EH, Upadhyay K, Talati AJ (2020). Comparison of institutional guidelines with established early onset sepsis risk calculator in reducing antibiotic use in an inner-city NICU in US. J Glob Antimicrob Resist.

[12] Chauhan N, Tiwari S, Jain U (2017). Potential biomarkers for effective screening of neonatal sepsis infections: an overview. Microb Pathog.

[13] Iroh Tam P, Bendel CM (2017). Diagnostics for neonatal sepsis: current approaches and future directions. Pediatr Res.

[14] Memar MY, Alizadeh N, Varshochi M, Kafil HS (2019). Immunologic biomarkers for diagnostic of early-onset neonatal sepsis. J Matern Fetal Neonatal Med.

[15] Sharma D, Farahbakhsh N, Shastri S, Sharma P (2018). Biomarkers for diagnosis of neonatal sepsis: a literature review. J Matern Fetal Neonatal Med.

[16] Eschborn S, Weitkamp JH (2019). Procalcitonin versus C-reactive protein: review of kinetics and performance for diagnosis of neonatal sepsis. J Perinatol.

[17] Chiesa C, Natale F, Pascone R, Osborn JF, Pacifico L, Bonci E, De Curtis M (2011). C reactive protein and procalcitonin: reference intervals for preterm and term newborns during the early neonatal period. Clin Chim Acta.

[18] Fukuzumi N, Osawa K, Sato I, Itawani S, Ishino R, Hayashi N, Iijima K, Saegusa J, Morioka I (2016). Age-specific percentile-based reference curve of serum procalcitonin concentrations in Japanese preterm infants. Sci Rep.

[19] Turner D, Hammerman C, Rudensky B, Schlesinger Y, Goia C, Schimmel MS (2006). Procalcitonin in preterm infants during the first few days of life: introducing an age related nomogram. Arch Dis Child Fetal Neonatal.

[20] Su H, Chang SS, Han CM, Wu KY, Li MC, Huang CY, Lee CL, Wu JY, Lee CC (2014). Inflammatory markers in cord blood or maternal serum for early detection of neonatal sepsis-a systemic review and meta-analysis. J Perinatol.

[21] Cantoni L, Ronfani L, Da Riol R, Demarini S, Perinatal Study Group of the Region Friuli-Venezia Giulia (2013). Physical examination instead of laboratory tests for most infants born to mothers colonized with group B Streptococcus: support for the Centers for Disease Control and Prevention’s 2010 recommendations. J Pediatr.

[22] Berardi A, Fornaciari S, Rossi C, Patianna V, Bacchi Reggiani ML, Ferrari F, Neri I, Ferrari F (2015). Safety of physical examination alone for managing well-appearing neonates ≥ 35 weeks’ gestation at risk for early-onset sepsis. J Matern Fetal Neonatal Med.

[23] Joshi NS, Gupta A, Allan JM, Cohen RS, Aby JL, Weldon B, Kim JL, Benitz WE, Frymoyer A (2018). Clinical monitoring of well-appearing infants born to mothers with chorioamnionitis. Pediatrics.

[24] Kuzniewicz MW, Walsh EM, Li S, Fischer A, Escobar GJ (2016). Development and implementation of an early-onset sepsis calculator to guide antibiotic management in late preterm and term neonates. Jt Comm J Qual Patient Saf.

[25] Escobar GJ, Puopolo KM, Wi S, Turk BJ, Kuzniewicz MW, Walsh EM, Newman TB, Zupancic J, Lieberman E, Draper D (2014). Stratification of risk of early-onset sepsis in newborns ≥ 34 weeks’ gestation. Pediatrics.

[26] Shakib J, Buchi K, Smith E, Young PC (2015). Management of newborns born to mothers with chorioamnionitis: is it time for a kinder, gentler approach?. Acad Pediatr.

[27] Kuzniewicz MW, Puopolo KM, Fischer A, Walsh EM, Li S, Newman TB, Kipnis P, Escobar GJ (2017). A quantitative, risk-based approach to the management of neonatal early onset sepsis. JAMA Pediatr.

[28] Money N, Newman J, Demissie S, Roth P, Blau J (2017). Antimicrobial stewardship: antibiotic use in well-appearing term neonates born to mothers with chorioamnionitis. J Perinatol.

[29] Warren S, Garcia M, Hankins C (2017). Impact of neonatal early onset sepsis calculator on antibiotic use within two tertiary healthcare centers. J Perinatol.

[30] Beavers JB, Bai S, Perry J, Simpson J, Peeples S (2018). Implementation and evaluation of the early-onset sepsis risk calculator in a high-risk university nursery. Clin Pediatr (Phila).

[31] Carola D, Vasconcellos M, Sloane A, McElwee D, Edwards C, Greenspan J, Aghai ZH (2018). Utility of early-onset sepsis risk calculator for neonates born to mothers with chorioamnionitis. J Pediatr.

[32] Dhudasia MB, Mukhopadhyay S, Puopolo KM (2018). Implementation of the sepsis risk calculator at an academic birth hospital. Hosp Pediatr.

[33] Gievers LL, Sedler J, Phillipi CA, Dukhovny D, Geddes J, Graven P, Chan B, Khaki S (2018). Implementation of the sepsis risk score for chorioamnionitis exposed newborns. J Perinatol.

[34] Klingaman C, King L, Neff-Bulger M (2018). Improved newborn care: evidence-based protocol for the evaluation and management of early-onset sepsis. Am J Med Qual.

[35] Strunk T, Buchiboyina A, Sharp M, Nathan E, Doherty D, Patole S (2018). Implementation of the neonatal sepsis calculator in an Australian tertiary perinatal centre. Neonatology.

[36] Akangire G, Simpson E, Weiner J, Noel-MacDonnell J, Petrikin J, Sheehan M (2020). Implementation of the neonatal sepsis calculator in early-onset sepsis and maternal chorioamnionitis. Adv Neonatal Care.

[37] Arora V, Strunk D, Furqan SH, Schweig L, Lefaiver C, George J, Prazad P (2019). Optimizing antibiotic use for early onset sepsis: a tertiary NICU experience. J Neonatal Perinatal Med.

[38] Bridges M, Pesek E, McRae M, Chabra S (2019). Use of an early onset-sepsis calculator to decrease unnecessary NICU admissions and increase exclusive breastfeeding. J Obstet Gynecol Neonatal Nurs.

[39] Eason J, Ward H, Danko O, Richardson K, Vaitkute R, McKeon-Carter R. Early-onset sepsis: can we screen fewer babies safely? Arch Dis Child. 2019. 10.1136/archdischild-2019-317047. 10.1136/archdischild-2019-317047 31678929

[40] Fowler NT, Garcia M, Hankins C (2019). Impact of integrating a neonatal early-onset sepsis risk calculator into the electronic health record. Pediatr Qual Saf.

[41] Goel N, Shrestha S, Smith R, Mehta A, Ketty M, Muxworthy H, Abelian A, Kirupaalar V, Saeed S, Jain S, Asokkumar A, Natti M, Barnard I, Pitchaikani PK, Banerjee S (2020). Screening for early onset neonatal sepsis: NICE guidance-based practice versus projected application of the Kaiser Permanente sepsis risk calculator in the UK population. Arch Dis Child Fetal Neonatal.

[42] Gong CL, Dasgupta-Tsinikas S, Zangwill KM, Bolaris M, Hay JW (2019). Early onset sepsis calculator-based management of newborns exposed to maternal intrapartum fever: a cost benefit analysis. J Perinatol.

[43] Hershkovich-Shporen C, Ujirauli N, Oren S, Juster Reicher A, Gadassi N, Guri A, Flidel-Rimon O. Not all newborns born to mothers with clinical chorioamnionitis need to be treated. J Matern Fetal Neonatal Med. 2019:1–6. 10.1080/14767058.2019.1651281. 10.1080/14767058.2019.1651281 31409159

[44] Joshi NS, Gupta A, Allan JM, Cohen RS, Aby JL, Kim JL, Benitz WE, Frymoyer A (2019). Management of chorioamnionitis-exposed infants in the newborn nursery using a clinical examination-based approach. Hosp Pediatr.

[45] Leonardi BM, Binder M, Griswold KJ, Yalcinkaya GF, Walsh MC (2019). Utilization of a neonatal early-onset sepsis calculator to guide initial newborn management. Pediatr Qual Saf.

[46] Stipelman CH, Smith ER, Diaz-ochu M, Spackman J, Stoddard G, Kawamoto K, Shakib JH (2019). Early-onset sepsis risk calculator integration into an electronic health record in the nursery. Pediatrics.

[47] Benincasa BC, Silveira RC, Paixão Schlatter R, Balbinotto Neto G, Procianoy RS (2020). Multivariate risk and clinical signs evaluations for early-onset sepsis on late preterm and term newborns and their economic impact. Eur J Pediatr.

[48] Morris R, Jones S, Banerjee S, Collinson A, Hagan H, Walsh H, Thornton G, Barnard I, Warren C, Reid J, Busfield A, Matthes J. Comparison of the management recommendations of the Kaiser Permanente neonatal early-onset sepsis risk calculator (SRC) with NICE guideline CG149 in infants ≥ 34 weeks’ gestation who developed early-onset sepsis. Arch Dis Child Fetal Neonatal Ed. 2020:fetalneonatal-2019-317165. 10.1136/archdischild-2019-317165. 10.1136/archdischild-2019-317165 32170032

[49] Perez EM, Taylor M, Swanson K, Laferney JD (2019). Implementation of an antibiotic stewardship quality improvement initiative in a community hospital for infants born at ≥35 weeks. Proc (Bayl Univ Med Cent).

[50] van der Weijden BM, Achten NB, Bekhof J, Evers EE, van Dongen O, Rijpert M, Kamps AWA, Ten Tusscher GW, van Houten MA, Plötz FB (2020). Neonatal early-onset sepsis calculator recommended significantly less empiric antibiotic treatment than national guidelines. Acta Paediatr.

